# Effects of Leucine-Enriched Whey Protein Supplementation on Physical Function in Post-Hospitalized Older Adults Participating in 12-Weeks of Resistance Training Program: A Randomized Controlled Trial

**DOI:** 10.3390/nu11102337

**Published:** 2019-10-01

**Authors:** Maria Amasene, Ariadna Besga, Iñaki Echeverria, Miriam Urquiza, Jonatan R. Ruiz, Ana Rodriguez-Larrad, Mikel Aldamiz, Pilar Anaut, Jon Irazusta, Idoia Labayen

**Affiliations:** 1 Department of Pharmacy and Food Science, University of the Basque Country UPV/EHU, 01006 Vitoria-Gasteiz, Spain; maria.amasene@ehu.eus; 2 Department of Medicine, Araba University Hospital, Bioaraba Research Institute, OSI Araba. CIBERSAM, University of the Basque Country (UPV/EHU), 01004 Vitoria-Gasteiz, Spain; mikel.aldamiz-echebarriasansebastian@osakidetza.eus (M.A.); mariapilar.anautmayo@osakidetza.eus (P.A.); 3 Department of Physiology, University of the Basque Country, UPV/EHU, 48940 Leioa, Spain; inaki.echeverriag@ehu.eus (I.E.); miriam.urquiza@ehu.eus (M.U.); ana.rodriguez@ehu.eus (A.R.-L.); jon.irazusta@ehu.eus (J.I.); 4 PROFITH “PROmoting FITness and Health through physical activity” Research Group, Sport and Health University Research Institute (iMUDS), Department of Physical Education and Sport, Faculty of Sport Sciences, University of Granada, 18071 Granada, Spain; ruizj@ugr.es; 5 ELIKOS group, Institute for Innovation and Sustainable Development in Food Chain (IS-FOOD), Public University of Navarra, 31006 Pamplona, Spain; idoia.labayen@unavarra.es

**Keywords:** elderly, aging, muscle mass, strength, resistance training, leucine, whey protein, protein supplementation

## Abstract

Age-related strength and muscle mass loss is further increased after acute periods of inactivity. To avoid this, resistance training has been proposed as an effective countermeasure, but the additional effect of a protein supplement is not so clear. The aim of this study was to examine the effect of a whey protein supplement enriched with leucine after resistance training on muscle mass and strength gains in a post-hospitalized elderly population. A total of 28 participants were included and allocated to either protein supplementation or placebo supplementation following resistance training for 12 weeks (2 days/week). Physical function (lower and upper body strength, aerobic capacity and the Short Physical Performance Battery (SPPB) test), mini nutritional assessment (MNA) and body composition (Dual X-ray Absorptiometry) were assessed at baseline and after 12 weeks of resistance training. Both groups showed improvements in physical function after the intervention (*p* < 0.01), but there were no further effects for the protein group (*p* > 0.05). Muscle mass did not improve after resistance training in either group (*p* > 0.05). In conclusion, 12 weeks of resistance training are enough to improve physical function in a post-hospitalized elderly population with no further benefits for the protein-supplemented group.

## 1. Introduction

Aging is characterized by a progressive decline in skeletal muscle mass and function defined as sarcopenia [1]. Sarcopenia is related to an increased risk of falling [1], fractures [2], physical disability [3] and mortality [4]. In healthy aging, muscle mass loss ranges from 3% to 8% per decade [5]. However, this decline is further emphasized by acute or chronic illness [6], inactivity [6] and inadequate protein and/or energy intake [7]. So, physical activity is proposed as an effective countermeasure to delay the age-related muscle mass loss [7]. Indeed, following a healthy lifestyle may help to prevent and reduce the consequences of age-related muscle mass loss [7].

A balanced protein metabolism is important to muscle mass accretion and maintenance [8]. Energy and protein intake are key nutritional factors to achieve protein balance [7]. However, due to several physiological and social factors, elderly people tend to reduce food intake and, in consequence, often fail to meet energy and protein requirements [9]. Likewise, protein-energy malnutrition is frequent in elderly patients [9]. Besides total daily protein intake [10], dietary protein quality and its anabolic potential have also received increased interest with the goal of optimizing skeletal muscle anabolism in the elderly [10,11]. 

Dietary protein quality depends on its digestibility, amino acid (AA) profile and AA availability [12,13]. Therefore, in studies aiming to optimize muscle mass among elderly people, whey protein (≈20 g/day) is considered superior to other isolated protein sources [14,15]. Whey protein is also characterized for being a high leucine-containing protein, which is the main precursor for activating muscle protein synthesis via mammalian target of rapamycin (mTOR) signaling [8,16]. A protein/AA source containing around 1.8–2.0 g of leucine would be enough to activate post-exercise “leucine trigger”, whereas in rested conditions, a higher dose might be required in young adults [16]. Other authors [15], reported that 20 g of whey protein enriched with 3 g of leucine post-exercise resulted in a greater muscle protein synthesis rate in healthy older people. 

Muscle mass accretion and strength gain depend on the synergistic effect of protein consumption and resistance training [8,16]. Protein ingestion close after exercise seems to increase exercise-induced muscle mass sensitivity to anabolism [8]. In a recent systematic review and meta-analysis [17], it was concluded that a combination of protein supplementation and resistance training led to positive effects on body composition, muscle volume and strength, and physical function in elderly people. In contrast, in people aged 70 years or older, it was shown that despite overall improvements from baseline for the majority of outcomes, there were no significant differences between the group receiving protein/AA supplementation along with resistance training and the group with resistance training alone [18]. 

Acute periods of inactivity, such as a hospital stay, accentuates age-related muscle mass loss [6]. After hospitalization, older individuals are more vulnerable to develop any adverse event [19]. Then, early interventions to accelerate recovery and avoid hospital readmission will be important [20]. For example, implementing interventions that combine nutrition and physical exercise immediately after discharge [20]. 

In view of this growing interest, the objective of the present study was to examine the effect of a whey protein supplement enriched with leucine after resistance training on muscle mass and strength gains in a post-hospitalized elderly population. We hypothesized that elderly people after hospitalization may benefit most from the synergetic effect of protein supplementation and a resistance training session. 

## 2. Methods

### 2.1. Study Design

The Sarcopenia and Fragilidad-protein (S and F-PROT) study is a prospective, 24-week, single-blind, randomized, placebo-controlled clinical trial (ClinicalTrials.gov ID: NCT03815201). The study was conducted at the facilities of the Araba University Hospital in Vitoria-Gasteiz (North Spain), from September 2017 to July 2018. The Clinical Research Ethics Committee of the Araba University Hospital (CEIC-HUA: 2017-021) approved the study protocol (S and F-PROT) that complied with the revised ethical guidelines of the Declaration of Helsinki (revision of 2013). All participants were informed about the details of the research and signed an informed consent before their enrolment in the study.

The S and F-PROT project compared relative changes on functional capacity (muscular strength of upper and lower limbs, and aerobic capacity), body composition (lean mass and fat mass at whole body, arms, legs and trunk) and nutritional status between two groups following a resistance training intervention program with post-exercise supplementation (Protein-group) or without supplementation (Placebo-group). 

### 2.2. Participants

**Figure 1:**
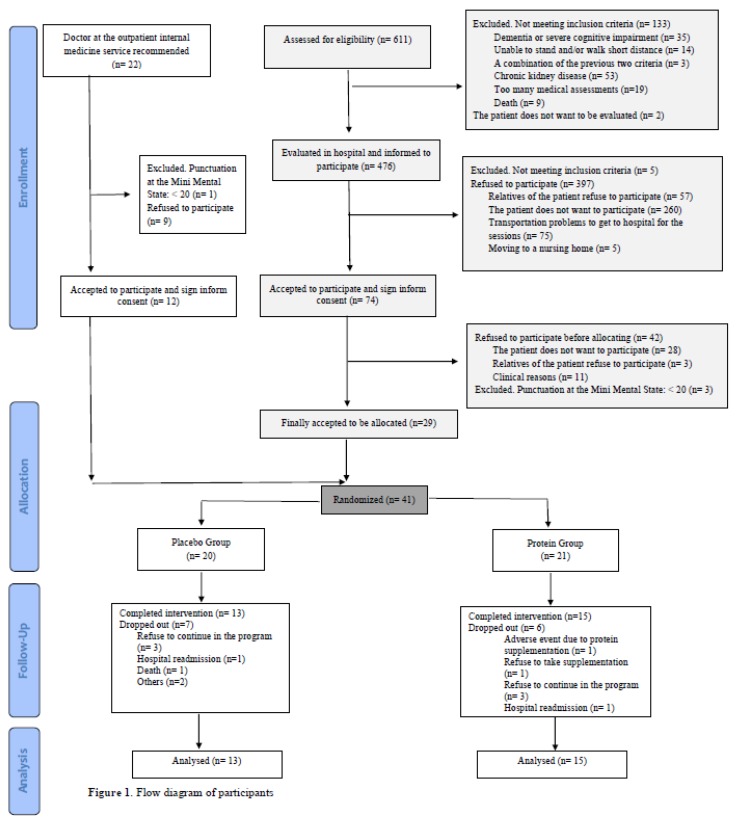
Flow Diagram of participants.

Volunteers accessed the program after hospitalization at the internal medicine service of the Araba University Hospital, or by medical recommendation at the outpatient internal medicine specialty at the Araba University Hospital (Figure 1). Hospitalized patients older than 70 years old were first pre-screened for eligibility. All pre-screened participants met the following criteria: >70 years old, a punctuation of ≥20 at the Mini Mental State Questionnaire (MMSE), fulfilled the criteria for sarcopenia diagnosis of the European Working Group on Sarcopenia in Older People, were able to walk alone or using a walking stick, a walking frame, or parallel walking bars, were able to understand the instructions or what had being said, and signed the informed consent. Patients were excluded for examination if they had any of the following exclusion criteria: history of chronic kidney disease, had suffered a heart attack in the last 3 months, been unable to walk, have suffered any fracture of the upper or lower limbs in the last 3 months, been suffering from severe dementia, a history of autoimmune neuromuscular disorders (for example, myasthenia gravis, Guillain–Barré syndrome, inflammatory myopathies) or amyotrophic lateral sclerosis, or refused to sign the informed consent.

Patients that were eligible for the intervention program were assessed for nutritional status (Mini Nutritional Assessment-Short Form (MNA-SF; Nestlé Nutrition Institute)) [21], physical function (Short Physical Performance Battery (SPPB) [22] and handgrip strength), frailty (a Spanish language version of the Fried test [23]) and cognitive function (Spanish validated version of the Pfeiffer test, the Short Portable Mental Status Questionnaire (SPMSQ) [24]) during their hospitalization. Patients were informed about the possibility of participating in an exercise training program after hospital discharge and an informed consent was given along with further written information. After a recovery week, patients were cited for baseline physical function assessment before initiating the intervention program. 

Many hospitalized patients did not meet inclusion criteria when assessing eligibility (21.8%) or refused to participate (66.6%) because of health issues, lack of interest in the physical exercise program, or had problems to get to hospital for the intervention sessions. As the hospital recruitment proved not to be enough for the intervention aims, the outpatient internal medicine service was chosen as an alternative recruitment source. Those patients at the outpatient internal medicine service potentially meeting inclusion criteria were informed by their doctor about the exercise intervention program. Thereafter, patients were cited for a first eligibility assessment with the investigation team. If participation criteria were met, patients were again cited a week after for baseline physical function assessment (Figure 1). 

### 2.3. Randomization

Following baseline physical function assessment, participants were randomly allocated to one of the two intervention groups: Placebo-group or Protein-group. Participant stratification was based on gender to ensure equal allocation in both groups.

### 2.4. Supplementation and Blinding

Placebo and protein supplements were delivered by the nutritionist in the first half hour following each training session. The protein supplement contained 20 g of whey protein isolate (Davisco ^®^: BiPRO all-natural whey protein isolate, Eden Paririe, MN, USA) enriched with 3 g of leucine (Nutricia, Madrid, Spain). The nutritional composition of both the placebo and protein supplement is shown in Table 1. The supplements were energy-matched and were flavored with lemon flavor and solubilized in 150 mL of water. Only participants were blinded for supplementation. Supplements were stored in boxes and only the research team could identify them. All supplements were developed, prepared and stored in boxes by Laboratorium Sanitatis SL (Tecnalia Research and Innovation, Vitoria-Gasteiz, Spain).

### 2.5. Design of the Resistance Training Program

Both groups followed a supervised resistance training program for 12 weeks. The program consisted of 1 h sessions on two non-consecutive days per week. The first week of intervention was used for familiarization, and 1-RM (repetition maximum) estimation by the individual’s functional capacity through Brzycki equation [25]. The load was then gradually increased during a month, and half exercises were performed at 50%–65% of the estimated 1-RM. During the subsequent months, load was increased until 70% of the estimated 1-RM was reached. Two sets were performed per exercise and load and maximum repetition for each exercise was personalized for each participant. All resistance training sessions were designed and supervised by a sport scientist with experience in resistance training for the elderly.

All training sessions started with warm-up exercises (heel stand, calf raises, chair stand exercise and neck movements) and were followed by strengthening exercises of upper and lower limbs (arm-curl exercise with the participant in a seated position and personalized load, knee extension exercise with personalized load in a seated position, standing knee flexion with personalized load, side hip raise, standing hip extension and chair stand exercise). In the same resistance training session, some exercises for dynamic balance improvement were also practiced (side-by-side stand, semi-tandem stand, tandem stand, monopodal stand, timed up and go, stepping around obstacles and step up and down exercises). The session finished with 5 min of cool-down, consisting mainly of stretching exercises.

### 2.6. Outcome Measures

Primary and secondary outcomes were assessed at baseline and after 12 weeks of intervention by the same trained researchers. Post-intervention measurements were scheduled within one week following the last exercise session. 

### 2.7. Primary Outcome: Physical Function 

Physical function was assessed at baseline and at week 13 (once supervised intervention period was finished). 

Physical function was assessed using a combination of tests. The tests used to assess lower and upper body strength and aerobic capacity were based on the Senior Fitness Test [26]. For lower and upper body strength, 30-Second Chair Stand Test and 30-Second Arm Curl Test were used, respectively. For upper body strength, isometric handgrip strength was also measured using a handled dynamometer (JAMAR^®^ PLUS + Hand dynamometer). Aerobic capacity was assessed by the 6 min walking test (6MWT). Hence, for physical function assessment, the SPPB test battery was also used [22]. This test includes the 4 m walking speed test, the standing balance test (side-by-side stand, semi-tandem stand and tandem stand) and the time to rise from a chair five times test [22].

### 2.8. Secondary Outcomes

#### 2.8.1. Nutritional Assessment

A nutritionist completed all nutritional questionnaires along with the participant and/or participant’s relative or caregiver. Participant’s nutritional status was assessed using the MNA questionnaire (Nestlé Nutritional Institute) [27]. This questionnaire contains 18 items divided into 4 categories: anthropometric assessment, general assessment, short dietary assessment and subjective assessment [27]. Each answer has a numerical value contributing to the final punctuation. A maximum of 30 points can be obtained. Punctuation ranging from 24 to 30 reflects normal nutritional status, from 17 to 23.5 risk of malnutrition and a punctuation under 17 reflects malnutrition [27]. 

#### 2.8.2. Body Composition

Body fat, lean mass, bone mass, bone mineral density (BMD) and bone mineral content (BMC) were assessed by dual-energy X-ray absorptiometry (DXA; HOLOGIC, QDR 4500). 

Body mass (OMRON HN-288, Digital Personal Scale, Barcelona, Spain) was measured barefoot following the standard protocols. Height was estimated using knee height determination (SECA 220, Hamburg, Germany) [28]. Body mass index (BMI) was calculated as body weight divided by height squared (kg/m^2^). Waist circumference, hip circumference, calf circumference and mid-arm circumference were measured with a nonelastic tape (CESCORF, Porto Alegre, Brasil) following the protocol recommended by the International Society for the Advancement of Kinanthropometry (ISAK). 

#### 2.8.3. Biochemical Parameters 

Biochemical parameters were obtained from fasting venous blood samples in Ethylenediaminetetraacetic acid -containing tubes and in serum tubes. These tubes were immediately carried to the laboratory and EDTA-containing tubes were centrifuged at 1000× g at 4 °C for 10 min, whereas serum tubes were centrifuged 90 min after blood collection at 1000× g at 20 °C for 15 min. Serum albumin, prealbumin and creatinine were measured as protein malnutrition markers. 

### 2.9. Statistical Analysis

Baseline characteristics between groups (i.e., placebo versus protein supplementation) were compared using independent Student’s *t*-test. 

Sample size estimation and power analysis was calculated for muscle mass increase. With a population size of 35 on each group, a significant alpha level of 0.05, and power >80%, the range for a statistically detectable change in muscle mass will be 1.5–2kg with a standard deviation of 1.5–1.7 kg. 

Data analysis was performed following the per-protocol principle. Changes in primary and secondary outcomes were calculated as Post-intervention *minus* Pre-intervention values. Differences between the placebo and the protein groups (fixed factor) in changes on primary outcomes and secondary outcomes were calculated by analyses of covariance adjusting with baseline values. 

All statistical analyses were performed using the statistical software SPSS version 20.0 (SPSS Inc., Chicago, IL, USA) with a level of significance of α = 0.05. Data are expressed as means ± standard error of the mean.

## 3. Results

During the recruitment period, a total of 476 hospitalized patients were evaluated and invited to participate in the study. From them, only 74 (15.5%) accepted to participate, whereas the remaining 402 patients refused to participate (83.4%) or did not meet inclusion criteria (1.15%). Finally, from the 74 patients who accepted to participate, a total of 29 participants (39.2%) were randomized for the study. Overall, 93.9% of the evaluated hospitalized patients did not participate in the study due to inclusion criteria or rejection to participate in the program. Regarding the recruitment from the outpatient internal medicine service, a total of 22 patients were recommended to participate, 40.9% of these patients refused to enter the intervention program or did not meet inclusion criteria (4.5%), whereas 54.54% accepted. In total, 41 patients were randomized for the intervention program, 20 entered the Placebo-group and 21 the Protein-group. From the allocated participants, 13 did not complete the 12 weeks of the intervention program (7 from the Placebo-group and 6 from the Protein-group). The main reason for dropping out from the study in both groups was that participants refused to continue in the program (15.0% of the randomized patients in the Placebo-group and 14.3% in the Protein-group). In the Protein-group, an adverse event was reported with protein supplementation regarding itchy throat and difficulties to inhale, whereas another participant refused to take the protein supplement, so both participants were dropped from the study (Figure 1). Baseline characteristics of the recruited participants can be found in Supplemental Table S1.

Table 2 shows baseline characteristics of participants. There were no statistically significant differences in body composition and nutritional status variables between groups at baseline. However, within the physical function parameters, the protein-group walked significantly more meters in the 6MWT at baseline (*p* < 0.05). In contrast, the Protein-group showed significantly greater lean mass on the legs (%) than the Placebo-group (*p* < 0.05). 

### 3.1. Effects of the Intervention on Primary Outcomes: Physical Function

Both groups showed improvements over time in all the physical function tests (*p* < 0.01), except for the handgrip strength test (Table 3). However, we did not observe any significant difference between groups in any of the measured physical function tests (Table 3). 

### 3.2. Effects of the Intervention on Secondary Outcomes 

We did not observe any significant difference on body composition measurements within groups at the end of the intervention, except for lean mass on arms within the protein-group (*p* < 0.05, Table 3). There were no significant differences in any of the body composition variables between the two groups (Table 3). 

The MNA scoring improved significantly within both groups after the intervention program (*p* < 0.05, Table 3). However, we did not observe any significant difference on changes in MNA score between groups (*p* < 0.5, Table 3).

Among serum markers of protein malnutrition, creatinine and albumin concentrations did not significantly change over time in either group (Table 3). Prealbumin concentrations significantly decreased in the Protein-group (*p* < 0.05, Table 3). Nevertheless, there were no significant differences on changes in protein nutritional status serum biomarkers between groups (Table 3). 

## 4. Discussion 

The current study aimed to examine the additional effect of a leucine-enriched protein supplementation on physical function, skeletal muscle mass and nutritional status after resistance training in a post-hospitalized elderly population. Results do not show further beneficial effects with protein and leucine-enriched supplementation after 12 weeks of resistance training (2 sessions/week) for any of the measured variables. These findings suggest that protein supplementation might not be determinant to see improvements in muscle mass and strength, and/or the time period of the intervention was not enough to see significant results. 

It is well-established that resistance training is an effective countermeasure to combat age-related skeletal muscle mass and strength loss [6,29]. It is proposed as a primary intervention for sarcopenia [30], frailty [31], malnutrition [32] and other geriatric syndromes [7]. Our results are in line with these guidelines, according to physical function measurements as both groups show improvement after resistance training. 

Resistance training stimulates muscle protein synthesis [33]. To take advantage of this anabolic stimuli, we considered protein supplementation as a complementary strategy following resistance training. In line with studies supporting this strategy [15,34], the protein-group received 20 g of whey protein enriched with 3 g of leucine after each session twice per week. However, there were no further benefits on physical function for the protein-group in this study. This is in contrast with some [34,35,36], but not all [37,38] previous studies. A recent systematic review [18], concluded that protein/AA supplementation did not further improve muscle strength in older subjects following a resistance training program. Nevertheless, both groups showed significant improvements in physical function parameters, except for handgrip strength. This result was also seen in the study carried out by Leenders et al. [29], where they suggested that handgrip strength is not a clinically relevant and/or valid measure to evaluate changes in muscle function in response to a resistance training program in the elderly. 

We observed no changes in body composition after the intervention in either group. Again, we did not see further benefits with protein supplementation. One previous study with participants aged 82 years reported a limited muscle plasticity that further limited strength gains in response to a progressive resistance training program [39]. So, our results in a population with the same average age (82 years) underscore the limited capacity to hypertrophy as we age [40]. Furthermore, when looking for studies regarding muscle mass and strength gains along with protein supplementation, among many of them, the target adult population are younger than age 80 [34,36,41,42,43]. The same issue can be seen in recent systematic reviews and meta-analyses, where most of the included studies are based in younger populations [44,45]. However, this blunted anabolic response might be overcome, or at least minimized, if adequate interventions are designed [46]. It seems that the protein synthesis capacity of the muscle is preserved up to very old age in response to anabolic stimuli [33]. 

There is still much controversy regarding protein supplementation in the elderly population due to poor compliance, high heterogeneity and underpowered studies evident from meta-analysis and systematic reviews [17,18,45]. Studies underlying protein supplementation as an effective measure to increase resistance training induced adaptations were based on short-term metabolic studies [15,34,47]. Conversely, dietary intervention studies, where long-term protein supplementation have been examined, have failed to observe measurable gains in skeletal muscle mass in the elderly population [35,37,38]. Tieland et al. supplemented the protein-group with 15 g of protein at breakfast and lunch for 24 weeks and they reported that protein intake in this group increased to more than 25 g with each meal (daily protein intake increased from 1.0 ± 0.1 to 1.4 ± 0.1g/kg body mass/day) [35]. However, they did not observe measurable gains in skeletal muscle mass for the protein-group as baseline protein intake was already high [35]. 

Contrary to Tieland et al. [35], participants in our study entered the interventional program after hospitalization where they had suffered an acute phase of illness and inactivity, and it would be reasonable to think that the protein-group should have benefit more from the resistance training along with protein supplementation. However, during the post-hospitalization period exists an acquired, transient period of vulnerability known as post-hospital syndrome, where the nutritional requirements are higher than normal to reverse this acute situation [19,48]. It has been suggested to increase dietary protein to 1.2–1.5 g/kg body mass/day during acute illness or up to 2.0 g/kg body mass/day in severe situations [48]. Thus, it could be speculated that if the protein supplementation protocol used in the study of Tieland et al. was not enough to see gains in muscle mass [35], and neither was the one applied in our study. It is probably that participants in our study were below the dietary protein recommendation set for acute phases or that the dietary treatment on this study did not increase the daily protein intake to have an effect. However, in contrast to Tieland et al. [35], the protein-group in our study was supplemented immediately after resistance training in order to overcome any daily protein deficiency and take advantage of the increased exercise-induced anabolic stimuli. In a recent study conducted in mobility-limited older adults, the protein-group was supplemented after resistance training with 20 g of whey protein three times per week for six months [42]. Englund et al. concluded that protein supplementation improved body composition [42]. However, the target population in the study conducted by Englund et al. had a total SPPB score of ≤9 [42], whereas the protein group in our study had a baseline total SPPB score of ≥9. This suggests that our participants were in better physical condition and that they had better body composition, although they entered the program after an acute illness phase. So, there might be different hypotheses to assess why further benefits were not seen with protein supplementation in muscle mass accretion. The acute illness phase might have been the limiting factor which had increased the nutritional requirements of our participants, not only for protein needs but also daily energy intake. So, it could be that until all nutritional requirements are met, just twice per week protein supplementation after resistance training is not enough for muscle mass accretion. Conversely, as baseline protein intake was not reported, it could be that it was already within the protein dietary recommendation and that our participants in the protein-group were, after all, within an acceptable range of physical and health condition or that the deviation for protein intake from baseline was not sufficient to induce muscle mass gains [49].

The latter hypothesis might be more probable because muscle mass did not increase with protein supplementation, but even more importantly, it did not decrease after resistance training in either group. This suggests that participants in this study were within the RDA for protein, otherwise negative protein balance would have occurred hampering muscle mass maintenance [50], and it also reinforces the idea that healthy elderly people with an adequate daily protein intake might not benefit from increased protein content [51]. Furthermore, at baseline, participants in the protein-group had an average punctuation of 24.5 in the MNA, which is considered a normal nutritional status. In turn, within the placebo-group, the average punctuation was 23.1, so they were almost at risk of malnutrition according to the MNA. But, following the resistance training program, the mean punctuation in the placebo-group increased to 25.3, achieving a good nutritional status. These results suggest that nutritional requirements were within an acceptable range among both groups leading to improved physical function variables and maintenance of muscle mass in both groups. Nevertheless, it is worth to mention that protein supplementation was not prescribed to participant body weight, which could benefit more muscle mass accretion. It is also important to highlight that the rate of protein turnover in older adults is slower, so improvements in strength and physical performance are often seen before measurable changes in skeletal muscle mass become apparent [48]. Thus, it is unlikely to observe significant changes in muscle mass after only 12 weeks of twice per week supervised intervention. For muscle mass accretion, a positive protein balance must be achieved over time along with resistance exercise [50]. Indeed, it might be that the overall volume employed in our resistance training program was not enough to see gains in muscle mass [52]. In addition, the sample size in this study might not be enough to see beneficial effects in muscle mass accretion following post-training protein supplementation [18,49,52]. 

### Limitations and Strength

The current study has several limitations. Daily protein and energy intake of participants were not controlled, we can merely speculate that probably total daily protein intake, the protein distribution among meals, the protein supplementation and/or the duration of our interventional program were not enough to increase muscle mass in this study. In addition, we assessed changes in body composition by DXA and with this methodology, differences smaller than 1.0 kg are not detectable [35]. The recruitment was lower than what we expected with only 20 and 21 participants in the Placebo-group and in the Protein-group, respectively. However, the dropout rate was high with only 13 and 15 participants completing the intervention study in the Placebo-group and in the Protein-group, respectively. When designing the study, it was proposed that 35 participants should be included to each group to see detectable improvements in muscle mass. So, this might be the main reason for not having seen significant improvements in muscle mass after the intervention. Another limitation is that the study was single-blinded and not double-blinded. One of the strengths of the study is that to the best of our knowledge, this is the first randomized study including post-hospitalized elderly adults in a resistance training program along with protein supplementation.

## 5. Conclusions

This study reinforces resistance training as a fundamental early intervention strategy to maintain muscle mass and increase gains in physical function parameters in post-hospitalized elderly adults. Thus, 12 weeks of supervised resistance training with a one-hour session over two days/week seems enough to enhance strength and physical function variables in post-hospitalized elderly adults. However, it does not clarify the additional benefits of a protein supplementation. 

The elderly population is a very heterogenic group, so future directions should focus on conducting studies among the different subgroups with special needs. There is a need to assess which might be the optimum length of an interventional study including resistance exercise and supplementation to induce gains in muscle mass and strength. Specifically, there is a growing interest in stablishing the characteristics of the best protein supplementation protocol and differences between healthy older adults and older adults with an acute or chronic disease and/or with one or more conditions of the geriatric syndrome. It would also be interesting for future studies to add muscle biopsies as direct measurements for muscle mass hypertrophy.

## Figures and Tables

**Table 1:** Nutritional composition of the protein and placebo supplements.

Nutritional Composition	Protein Supplement
Β-lactoglobulin (g/bottle)	20
L-Leucine (g/bottle)	3
Sodium saccharin (g/bottle)	0.050
Sucralose (g/bottle)	0.030
Lemon flavor 654500 (g/bottle)	0.250
	Placebo supplement
Maltodextrin (g/bottle)	23
Hydroxyethylcellulose (g/bottle)	0.200
Lemon flavor 654500 (g/bottle)	0.250

**Table 2:** Characteristics of participants completing the study (intend-to-treat analyses).

	N	Placebo Group	N	Protein Group	*p*
Age (years)	13	81.7 (6.45)	15	82.9 (5.59)	0.607
Women (N, %)	13	7 (53.8)	15	7 (46.7)	0.717
Body mass (kg)	13	75.9 (17.95)	15	68.0 (11.43)	0.188
BMI (Kg/m^2^)	13	30.8 (6.53)	15	27.4 (3.50)	0.110
*Physical Function*					
Handgrip (kg/body mass)	13	0.3 (0.09)	15	0.4 (0.09)	0.063
SFT chair stand test 30sec	13	10.6 (4.17)	15	12.3 (2.97)	0.229
SFT arm curl test 30sec	13	13.5 (5.22)	15	16.3 (3.92)	0.137
SFT 6MWT (m)	13	314.8 (139.36)	15	411.5 (80.40)	0.040
SPPB total punctuation	13	8.7 (2.36)	15	10.1 (1.58)	0.089
SPPB 5Squat	13	14.7 (6.85)	15	12.2 (2.86)	0.232
*Body composition*					
Waist to hip ratio	13	1.00 (0.07)	15	0.98 (0.09)	0.459
Lean mass arms (kg)	13	2.3 (0.67)	15	2.3 (0.44)	0.897
Lean mass legs (kg)	13	6.8 (1.70)	15	6.4 (1.08)	0.441
Lean mass trunk (kg)	13	23.0 (4.83)	15	21.5 (3.89)	0.380
Total lean mass (kg)	13	45.2 (9.85)	15	42.3 (6.63)	0.391
Fat mass arms (%)	13	2.6 (0.96)	15	2.4 (0.77)	0.545
Fat mass legs (%)	13	5.8 (1.85)	15	5.4 (1.84)	0.603
Fat mass trunk (%)	13	17.1 (3.87)	15	14.9 (3.03)	0.124
Total fat mass (%)	13	35.4 (8.05)	15	32.1 (6.84)	0.259
*Nutritional Status*					
MNA score	13	23.1 (3.82)	15	24.5 (2.11)	0.273
Normal nutritional status (N, %)	13	4 (30.8)	15	11 (73.3)	0.064
At risk of malnutrition (N, %)	13	8 (61.5)	15	4 (26.7)	
Malnourished (N, %)	13	1 (7.7)	15	0 (0)	
Biomarkers					
Creatinine (mg/dL)	10	1.1 (0.48)	15	0.9 (0.35)	0.401
Albumin (g/dL)	13	4.0 (0.39)	15	4.0 (0.31)	0.994
Prealbumin (mg/dL)	12	22.2 (6.63)	14	23.3 (4.31)	0.613

BMI: body mass index; MNA score: Mini Nutritional Assessment score; SFT chair stand test 30 s: Senior Fitness Test chair stand test 30 s; SFT arm curl test 30 s: Senior Fitness Test arm curl test 30 s; SFT 6MWT (m): Senior Fitness Test 6-min Walking Test (m); SPPB total punctuation: Short Physical Performance Battery total punctuation; SPPB 5Squat: Short Physical Performance Battery 5Squat. Values are means and standard deviations.

**Table 3:** Body composition, nutritional status and physical function in elderly patients before (Pre) and after (Post) their participation in the resistance exercise intervention program plus protein supplementation (Protein-group) or placebo (placebo-group) (analyses per protocol).

	Placebo-Group				Protein-Group				Differences between Groups		
	N	Pre	Post	*p*	N	Pre	Post	*p*	Δ Placebo	Δ Protein	*p*
**Primary outcome**											
*Physical function*											
Handgrip (kg/body mass)	13	0.3 (0.09)	0.3 (0.09)	0.775	15	0.4 (0.09)	0.4 (0.09)	0.651	0.0 (0.03)	-0.0 (0.06)	0.971
SFT chair stand test 30sec	13	10.6 (4.17)	13.5 (4.59)	0.003	15	12.3 (2.97)	14.4 (3.22)	<0.001	2.8 (2.79)	2.1 (1.53)	0.480
SFT arm curl test 30sec	13	13.5 (5.22)	21.9 (4.66)	<0.001	15	16.3 (3.92)	23.5 (4.53)	<0.001	8.4 (5.74)	7.2 (4.86)	0.724
SFT 6min WT (m)	13	314.8 (139.36)	375.0 (128.39)	0.002	15	411.5 (80.4)	455.1 (81.77)	0.005	60.2 (53.67)	43.6 (51.2)	0.959
SPPB total score	13	8.7 (2.36)	10.3 (1.89)	0.001	15	10.1 (1.58)	11.3 (0.96)	0.002	1.6 (1.39)	1.2 (1.21)	0.634
SPPB 5Squat	13	14.7 (6.85)	10.6 (3.67)	0.005	15	12.2 (2.86)	10.0 (2.81)	0.004	−4.1 (4.32)	–2.2 (2.4)	0.491
**Secondary outcomes**											
*Body composition*											
Body mass (kg)	13	75.9 (17.95)	75.6 (18.31)	0.621	15	68.0 (11.43)	68.3 11.07)	0.500	−0.3 (2.24)	0.3 (1.60)	0.471
BMI (kg/m^2^)	13	30.8 (6.54)	30.7 (6.64)	0.575	15	27.4 (3.5)	27.5 (3.37)	0.453	−0.3 (2.24)	0.3 (1.60)	0.493
Waist to hip ratio	13	1.00 (0.07)	1.00 (0.08)	0.818	15	0.98 (0.09)	0.96 (0.08)	0.255	−0.0 (0.06)	−0.0 (0.05)	0.400
Lean mass arms (kg)	13	2.3 (0.67)	2.3 (0.41)	0.937	15	2.3 (0.44)	2.2 (0.41)	0.049	0.0 (0.36)	−0.1 (0.24)	0.088
Lean mass legs (kg)	13	6.8 (1.7)	6.9 (1.45)	0.630	15	6.4 (1.08)	6.5 (1.04)	0.260	0.1 (0.64)	0.1 (0.34)	0.756
Lean mass trunk (kg)	13	23.0 (4.83)	22.6 (4.47)	0.212	15	21.5 (3.88)	21.7 (3.61)	0.198	−0.4 (1.21)	0.2 (0.67)	0.128
Total lean mass (kg)	13	45.2 (9.85)	44.7 (8.54)	0.545	15	42.3 (6.63)	42.5 (6.61)	0.458	−0.4 (2.52)	0.2 (1.02)	0.611
Fat mass arms (%)	13	2.6 (0.96)	2.6 (0.85)	0.808	15	2.4 (0.77)	2.3 (0.92)	0.291	−0.0 (0.56)	−0.1 (0.41)	0.575
Fat mass legs (%)	13	5.8 (1.85)	5.9 (2.07)	0.165	15	5.4 (1.84)	5.5 (1.69)	0.506	0.2 (0.45)	0.1 (0.46)	0.549
Fat mass trunk (%)	13	17.1 (3.86)	16.7 (3.31)	0.448	15	14.9 (3.03)	15.7 (2.61)	0.061	−0.4 (1.86)	0.7 (1.31)	0.297
Total fat mass (%)	13	35.4 (8.05)	35.2 (7.53)	0.728	15	32.1 (6.84)	32.7 (6.64)	0.092	−0.2 (1.91)	0.6 (1.31)	0.357
*Nutritional status*											
MNA score	13	23.1 (3.8)	25.3 (2.2)	0.010	15	24.5 (2.1)	26.2 (1.6)	0.019	2.2 (2.6)	1.7 (2.5)	0.512
Normal nutritional status (N. %)	13	4(30.8)	9(69.3)	0.123	15	11(73.3)	14(93.4)	0.533			
At risk of malnutrition (N. %)	13	8(61.6)	4(30.8)		15	4(26.7)	1(6.7)				
Malnourished (N. %)	13	1(7.7)	0		15	0	0				
Biomarkers											
Creatinine (mg/dL)	10	1.1 (0.48)	1.1 (0.37)	0.664	15	0.9 (0.35)	0.9 (0.32)	0.595	0.0 (0.21)	0.0 (0.14)	0.438
Albumin (g/dL)	13	3.9 (0.39)	4.1 (0.31)	0.189	15	3.9 (0.31)	4.0 (0.26)	0.499	0.1 (0.22)	0.0 (0.15)	0.331
Prealbumin (mg/dL)	12	22.2 (6.63)	20.5 (4.48)	0.221	14	23.3 (4.31)	21.3 (4.17)	0.019	−1.6 (4.36)	−1.9 (2.77)	0.916

SFT chair stand test 30sec: Senior Fitness Test chair stand test 30 sec; SFT arm curl test 30 sec: Senior Fitness Test arm curl test 30sec; SFT 6MWT (m): Senior Fitness Test 6-min Walking Test (m); SPPB total punctuation: Short Physical Performance Battery total punctuation; SPPB 5Squat: Short Physical Performance Battery 5Squat; BMI: body mass index; MNA score: Mini Nutritional Assessment score. Values are means and standard deviations. *P indicates statistical differences between Pre and Post values (paired Student’s *t*-test). Δ placebo indicates the difference between Pre and Post values in the Placebo-group; Δ Protein indicates the difference between Pre and Post values in the Protein-group. *P* indicates statistical significance between Δ placebo and Δ Protein (ANOVA).

## Supplementary Materials

The following are available online at https://www.mdpi.com/2072-6643/11/10/2337/s1, Table S1: Characteristics of the recruited participants in the study (intend-to-treat analyses).
