# Supplementary material for: Effects of Leucine-Enriched Whey Protein Supplementation on Physical Function in Post-Hospitalized Older Adults Participating in 12-Weeks of Resistance Training Program: A Randomized Controlled Trial
Source: Nutrients. 2019 Oct 1;11(10):2337. doi: 10.3390/nu11102337 (PMC6835698; doi:10.3390/nu11102337)
Supplement: Supplementary file 1 [file nutrients-11-02337-s001.pdf]

Supplemental Table S1. Characteristics of the recruited participants in the study (*intend-to-treat* analyses).

	N	Total	N	Placebo group	N	Protein group	P
Age (years)	41	82.1(5.89)	20	81.2 (6.14)	21	82.9 (5.67)	0.354
Women (N, %)	41	22 (53.7)	20	10 (50)	21	12 (57.1)	0.647
Body mass (kg)	40	72.4 (15.6)	19	77.5 (17.02)	21	67.8 (12.92)	0.052
BMI (Kg/m^2)	40	29.1 (5.22)	19	31.1 (5.83)	21	27.4 (3.95)	0.025
Physical Function							
Handgrip (kg/body mass)	40	0.3 (0.09)	19	0.3 (0.09)	21	0.4 (0.09)	0.021
SFT chair stand test 30sec	41	10.6 (4.39)	20	9.9 (4.91)	21	11.2 (3.86)	0.358
SFT arm curl test 30sec	41	14.9 (4.98)	20	14.5 (5.09)	21	15.4 (4.95)	0.557
SFT 6MWT (m)	41	352.1 (119.45)	20	315.3 (131.26)	21	387.2 (97.61)	0.056
SPPB total punctuation	41	9.1 (2.4)	20	8.7 (2.4)	21	9.5 (2.36)	0.278
SPPB 5Squat	38	13.2 (4.99)	18	13.7 (6.17)	20	12.8 (3.76)	0.602
Body composition							
Waist to hip ratio	40	0.99 (0.09)	19	1.01 (0.07)	21	0.97 (0.1)	0.120
Lean mass arms (kg)	41	2.4 (0.63)	20	2.5 (0.75)	21	2.3 (0.49)	0.310
Lean mass legs (kg)	41	6.7 (1.43)	20	6.9 (1.58)	21	6.5 (1.27)	0.382
Lean mass trunk (kg)	41	22.2 (4.39)	20	23.2 (4.73)	21	21.2 (3.91)	0.142
Total lean mass (kg)	41	43.9 (8.62)	20	45.8 (9.68)	21	42.2 (7.27)	0.189
Fat mass arms (%)	40	2.5 (0.91)	19	2.7 (0.93)	21	2.3 (0.88)	0.170
Fat mass legs (%)	40	5.8 (1.74)	19	5.9 (1.72)	21	5.6 (1.78)	0.499
Fat mass trunk (%)	40	15.9 (3.53)	19	17.3 (3.28)	21	14.5 (3.29)	0.011
Total fat mass (%)	40	33.8 (7.39)	19	36.1 (7.25)	21	31.8 (7.08)	0.066
Nutritional Status							
MNA score	39	23.5 (3.0)	19	23.0 (3.5)	20	24.0 (2.4)	0.311
Normal nutritional status (N, %)	39	20 (48.7)	19	7 (35)	20	13 (61.9)	0.160
At risk of malnutrition (N, %)	39	18 (43.9)	19	11 (55)	20	7 (33.3)	
Malnourished (N, %)	39	1 (2.4)	19	1 (5)	20	0 (0)	
Biomarkers							
Creatinine (mg/dl)	37	1.0 (0.39)	17	1.1 (0.46)	20	0.9 (0.33)	0.228
Albumin (g/dl)	40	4.0 (0.32)	20	4.0 (0.36)	20	4.0 (0.28)	0.806
Prealbumin (mg/dl)	37	23.0 (5.52)	18	23.6 (6.48)	19	22.5 (4.54)	0.535

BMI: body mass index; MNA score: Mini Nutritional Assessment score; SFT chair stand test 30sec: Senior Fitness Test chair stand test 30sec; SFT arm curl test 30sec: Senior Fitness Test arm curl test 30sec; SFT 6MWT (m): Senior Fitness Test 6-minute Walking Test (m); SPPB total punctuation: Short Physical Performance Battery total punctuation; SPPB 5Squat: Short Physical Performance Battery 5Squat.
